# Is the use of electronic cigarettes while smoking associated with smoking cessation attempts, cessation and reduced cigarette consumption? A survey with a 1‐year follow‐up

**DOI:** 10.1111/add.12917

**Published:** 2015-04-23

**Authors:** Leonie S. Brose, Sara C. Hitchman, Jamie Brown, Robert West, Ann McNeill

**Affiliations:** ^1^ Department of Addictions, UK Centre for Tobacco and Alcohol Studies (UKCTAS), Institute of Psychiatry, Psychology and NeuroscienceKing's College LondonLondonUK; ^2^ Health Behaviour Research CentreUniversity College LondonLondonUK

**Keywords:** Electronic cigarettes, electronic nicotine delivery systems, harm reduction, smoking cessation, tobacco, quit attempts

## Abstract

**Aims:**

To use a unique longitudinal data set to assess the association between e‐cigarette use while smoking with smoking cessation attempts, cessation and substantial reduction, taking into account frequency of use and key potential confounders.

**Design:**

Web‐based survey, baseline November/December 2012, 1‐year follow‐up in December 2013.

**Setting:**

Great Britain.

**Participants:**

National general population sample of 4064 adult smokers, with 1759 (43%) followed‐up.

**Measurements:**

Main outcome measures were cessation attempt, cessation and substantial reduction (≥50% from baseline to follow‐up) of cigarettes per day (CPD). In logistic regression models, cessation attempt in the last year (analysis *n* = 1473) and smoking status (*n* = 1656) at follow‐up were regressed on to baseline e‐cigarette use (none, non‐daily, daily) while adjusting for baseline socio‐demographics, dependence and nicotine replacement (NRT) use. Substantial reduction (*n* = 1042) was regressed on to follow‐up e‐cigarette use while adjusting for baseline socio‐demographics and dependence and follow‐up NRT use.

**Findings:**

Compared with non‐use, daily e‐cigarette use at baseline was associated with increased cessation attempts [odds ratio (OR) = 2.11, 95% confidence interval (CI) = 1.24–3.58, *P* = 0.006], but not with cessation at follow‐up (OR = 0.62, 95% CI = 0.28–1.37, *P* = 0.24). Non‐daily use was not associated with cessation attempts or cessation. Daily e‐cigarette use at follow‐up was associated with increased odds of substantial reduction (OR = 2.49, 95% CI = 1.14–5.45, *P* = 0.02), non‐daily use was not.

**Conclusions:**

Daily use of e‐cigarettes while smoking appears to be associated with subsequent increases in rates of attempting to stop smoking and reducing smoking, but not with smoking cessation. Non‐daily use of e‐cigarettes while smoking does not appear to be associated with cessation attempts, cessation or reduced smoking.

## Introduction

In electronic cigarettes, a battery‐powered heating element heats a solution, usually containing nicotine, to produce a aerosol. The use of e‐cigarettes has increased dramatically in the last few years; users are almost exclusively smokers or former smokers, with fewer than 1% of never‐smokers using them regularly 1, 2, 3, 4, 5, 6, 7, 8. The vast majority of e‐cigarette users report using them to stop smoking tobacco 6, 9 and in England, for example, smokers attempting to stop smoking now use e‐cigarettes more often than any other aid, including nicotine replacement therapy (NRT) 10. Smoking prevalence in England has been declining from 20% in 2012 to 18.4% in 2014 (up to October), and in 2014 smoking cessation rates were the highest since at least 2008 10, 11. This simultaneous increase in e‐cigarette use and cessation may be coincidental, and it is therefore vitally important for longitudinal studies to be conducted to assess the impact of e‐cigarette usage on quitting behaviour.

Evidence on NRT supports the possibility of a link between using e‐cigarettes that deliver nicotine and attempts to stop smoking. Use of NRT while smoking is associated with a small reduction in cigarette consumption and a significant increase in the likelihood of subsequent smoking cessation even in smokers without intentions to stop smoking 12, 13. Very little evidence is available to evaluate whether a similar pattern is observed with use of e‐cigarettes by smokers and only a handful of studies have used any longitudinal data on e‐cigarette use and smoking behaviour. A trial in smokers not intending to quit compared e‐cigarettes with no nicotine with e‐cigarettes with two different nicotine strengths and found that all led to significant reduction in tobacco consumption, and that significantly more smokers using the e‐cigarettes with nicotine quit smoking 14. In a web‐based survey of a national sample of current smokers in the United States who were followed‐up 1 year later, e‐cigarette use at baseline did not predict smoking cessation 1 year later 15. Data from two waves of the International Tobacco Control survey showed that smokers who were using e‐cigarettes at follow‐up were more likely to have reduced their cigarette consumption than non‐users, but cessation did not differ 9. Among a cohort of young adults in the United States, those who had used e‐cigarettes at least once in the month before baseline had a similar likelihood of quitting smoking 1 year later to those who had never used e‐cigarettes 16. Unfortunately, none of these analyses distinguished frequency of use and many defined any trial or experimentation, even if just once, as use, so it is unclear what proportion were actually using e‐cigarettes with any regularity. Regular use is likely to have a stronger effect on smoking behaviour than trial or infrequent use. When separating regular from intermittent use, respondents who had used e‐cigarettes daily for at least a month were far more likely to have quit smoking than those who had not used them, whereas there was no such association of quitting with intermittent e‐cigarette use 17. This highlights the importance of disentangling use from trial; however, the intensity of e‐cigarette use had to be determined retrospectively. Because use is more common in smokers making quit attempts and all those who had quit must have made a quit attempt, this method confounds e‐cigarette use with quit attempts.

To address the question as to whether use of e‐cigarettes by smokers is associated with smoking behaviour change, this study used a web‐based national sample from the general population in Great Britain with a 1‐year follow‐up.

We used the two waves of survey data to assess the association of:

1. daily, non‐daily and non‐use of e‐cigarettes in smokers at baseline with smoking cessation attempts during follow‐up (quit attempt analysis);
2. daily, non‐daily and non‐use of e‐cigarettes in smokers at baseline with smoking cessation at follow‐up (cessation analysis); and
3. daily, non‐daily and non‐use of e‐cigarette use at follow‐up with substantial reduction in tobacco cigarette consumption from baseline to follow‐up. First (primary reduction analysis), we excluded those using e‐cigarettes at baseline because, if use of e‐cigarettes is associated with reduction in tobacco consumption, respondents may already have reduced their consumption at baseline, making detection of reduction from baseline to follow‐up less likely. As it could also be argued that e‐cigarette using smokers should be reducing further, we then also included smokers using e‐cigarettes at both time‐points (secondary reduction analysis).

## Methods

### Design

This was a web‐based longitudinal survey, with baseline data collected in November/December 2012 and follow‐up in December 2013. University College London ethics committee confirmed that specific approval was not required. Data were anonymized before being passed to the research team.

### Sample

**Figure 1:**
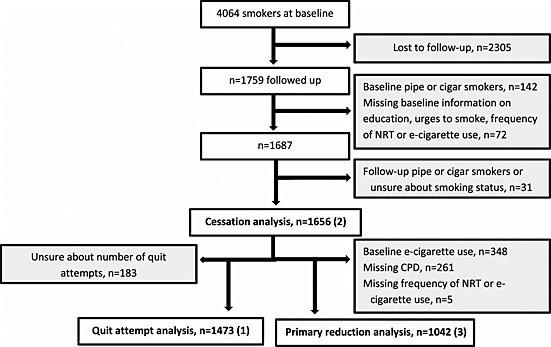
Sample flowchart. Grey boxes indicate exclusions. Bold numbers in brackets indicate the three different outcomes. CPD = cigarettes per day

The study sample was recruited from an online panel managed by Ipsos MORI. Ipsos MORI is the second largest market research organization in the United Kingdom. Members were invited by e‐mail to participate in an online study about smoking. By completing the survey respondents would earn points which could be redeemed against high street vouchers or used to enter a prize draw. Each respondent logged into their Ipsos MORI online account and was asked a screening question about their past‐year smoking status. Between November and December 2012, a total of 23 785 respondents were asked the screening question of whom 25.9% (*n* = 6165) had smoked in the past year. This proportion was similar to that identified by a face‐to‐face survey of representative samples of the population in England during 2012 10. Five thousand respondents completed the survey (4064 current smokers). They were re‐contacted 1 year later for follow‐up. Follow‐up achieved a response rate of 43.6% overall (*n* = 2182) and of 43.3% among baseline smokers (*n* = 1759). Figure 1 shows the selection of analyses samples for the three main outcomes.

The secondary reduction analysis included smokers using e‐cigarettes at both time‐points (*n* = 1005).

### Measures

Baseline and follow‐up surveys included a range of questions on socio‐demographic and smoking characteristics, nicotine use, quit attempts and health status. The current analyses included the following measures, fully presented in the Supporting information, Appendix.

#### Outcome measures

1. Quit attempts: smokers and recent ex‐smokers were asked about the number of attempts to stop they had made in the previous year. Those reporting at least one attempt and 37 respondents who did not report an attempt but had stopped smoking between baseline and follow‐up were coded as having made an attempt.

Cessation: smoking status was assessed at baseline and follow‐up in all respondents. Change from being a smoker at baseline to being an ex‐smoker at follow‐up was coded as cessation.

3 Substantial reduction: smoking characteristics included the number of cigarettes smoked per day (CPD) for daily smokers and the number of cigarettes per week for non‐daily smokers. Number of cigarettes per week were divided by seven to calculate CPD. Substantial reduction was defined as a reduction by at least 50% from baseline CPD to follow‐up CPD 13.

#### Socio‐demographic characteristics, dependence and nicotine use

All characteristics were measured at baseline and follow‐up; the Analysis section explains which time‐points were used in each analysis. Respondents provided their age, gender and highest level of formal education (see Supporting information, Appendix for questions and response options). Level of education was collapsed into those with any university education (including ‘some university’) and those without university education.

Strength of urges to smoke (SUTS) can be used as a measure of dependence and is a strong predictor of successful cessation in population samples 18, 19. The SUTS was included rather than the Fagerstrom Test of Nicotine Dependence (FTND 20) or the subset of FTND questions used for the Heaviness of Smoking Index (HSI 21) for two reasons. One reason was that the SUTS has outperformed the FTND in predicting failure of quit attempts 18; the second was that we hypothesized e‐cigarette use to have an effect on smoking behaviour, specifically on the number of cigarettes smoked, one of the two components of the HSI, which would limit the comparability of scores across users and non‐users of e‐cigarettes.

Smokers and recent ex‐smokers also reported if they were using NRT for any reason (not necessarily for a quit attempt), and how frequently they used NRT products. Respondents who had heard of e‐cigarettes were asked whether they had ever tried one and, if they had, how often they were currently using an e‐cigarette. For the main analyses, frequency of use of NRT and e‐cigarettes were each collapsed into daily, non‐daily and none.

### Analysis

Respondents who completed the follow‐up were compared with those who did not respond to the invitation in terms of socio‐demographic characteristics, nicotine use and dependence using *t*‐tests or analyses of variance (ANOVAs) for continuous data and χ^2^ statistics for categorical data.

In the main logistic regression models, reports of at least one quit attempt in the last year and smoking status at follow‐up were regressed onto baseline e‐cigarette use (none, non‐daily, daily) while adjusting for baseline age, gender, education, dependence (SUTS) and NRT use. Similar logistic regression models were used to analyse substantial reduction in CPD, but using NRT and e‐cigarette use at follow‐up, not baseline. Because only a small number of respondents overall had reduced substantially and 26.1% (*n* = 322) of the sample for the primary reduction analysis had increased consumption, the quantitative change in CPD was analysed using multiple linear regression, adjusting for the same characteristics as in the logistic regressions but dummy‐coding NRT and e‐cigarette use.

As sensitivity analyses, we collapsed daily and non‐daily e‐cigarette use categories and conducted logistic regressions using the collapsed variable while adjusting as in the main models.

SPSS version 21 was used for all analyses.

## Results

Prevalence and characteristics of users of e‐cigarettes in the baseline survey have been reported previously 22. In brief, more than 90% of current smokers and recent ex‐smokers were aware of e‐cigarettes, approximately a third had ever used e‐cigarettes and a fifth was currently using them. Daily use was more common in recent ex‐smokers (46% of current users) than in current smokers (23%). Age and gender split did not differ between users and non‐users. Among smokers, e‐cigarette users had a higher socio‐economic status than non‐users and were more likely to have made a quit attempt in the past year. Users reported higher tobacco cigarette consumption than non‐users 22.

Follow‐up respondents differed from respondents lost to follow‐up on some baseline characteristics. Those lost to follow‐up were younger and women were more likely to be lost to follow‐up than men. Frequency of NRT use differed; those who used NRT less than daily were more probably lost to follow‐up (Supporting information, Table S1). Education, dependence and frequency of e‐cigarette use did not differ.

A range of e‐cigarettes were used and will be reported in a separate publication 23; briefly, a majority used ‘first generation’ e‐cigarettes that were cigarette‐like in appearance (‘cigalikes’).

### Quit attempts

Overall, 46.2% (*n* = 680) of respondents in the analysis made a quit attempt; 43.7% (*n* = 508) of non‐users of e‐cigarettes, 52.5% (*n* = 124) of non‐daily e‐cigarette users and 64.9% (*n* = 48) of daily users. Sample characteristics are presented in Table 1. In unadjusted analysis, both daily [odds ratio (OR) = 2.38, 95% confidence interval (CI) = 1.46–3.89, *P* = 0.001] and non‐daily e‐cigarette use (OR = 1.43, 95% CI = 1.08–1.89, *P* = 0.013) were associated with increased likelihood of quit attempts compared with non‐use.

**Table 1:** Logistic regression analyses of association of baseline socio‐demographics, dependence [strength of urges to smoke (SUTS)] and non‐cigarette nicotine intake with quit attempts and smoking cessation during follow‐up.

		*Quit attempt (n = 1473, of whom n = 680 made attempt)*				*Cessation (n = 1656, of whom n = 200 stopped smoking)*			
		*n(%)/mean (SD)*	*OR*	*95% CI*	*P*	*n(%)/mean (SD)*	*OR*	*95% CI*	*P*
Age^a^		46.6 (15.2)	0.83	0.77–0.90	<0.001	45.7 (15.3)	0.88	0.79–0.97	0.009
Gender	Female	642 (43.6)	1			720 (43.5)	1		
	Male	831 (56.4)	0.84	0.67–1.05	0.12	936 (56.5)	0.86	0.64–1.16	0.32
Level of education	No HE	958 (65.0)	1			1074 (64.9)	1		
	Some HE	515 (35.0)	0.83	0.66–1.05	0.12	582 (35.1)	0.76	0.55–1.05	0.099
SUTS^b^		2.2 (1.1)	1.06	0.96–1.18	0.25	2.2 (1.1)	0.74	0.64–0.86	<0.001
NRT use	None	1212 (82.3)	1			1339 (80.9)	1		
	Non‐daily	161 (10.9)	4.21	2.89–6.14	<0.001	193 (11.7)	1.39	0.88–2.21	0.16
	Daily	100 (6.8)	9.43	5.17–17.23	<0.001	124 (7.5)	1.67	0.98–2.84	0.062
E‐cig use	None	1163 (79.0)	1			1307 (78.9)	1		
	Non‐daily	236 (16.0)	1.18	0.87–1.60	0.29	263 (15.9)	0.77	0.49–1.21	0.25
	Daily	74 (5.0)	2.11	1.24–3.58	0.006	86 (5.2)	0.62	0.28–1.37	0.24

a Mean and standard deviation (SD) presented, odds ratios (OR) for single year raised to the power of 10 to present per 10‐year increase.

b Strengths of urges to smoke, possible range 0 ‘no urges’ to 5 ‘extremely strong urges’, mean and SD presented, OR per unit increase. HE = higher education; NRT = nicotine replacement therapy.

While adjusting for socio‐demographic characteristics, dependence and NRT use, daily e‐cigarette use at baseline was associated with increased odds of making an attempt to stop smoking compared with non‐use. Non‐daily e‐cigarette users did not differ significantly from non‐users (Table 1). There was a strong association of quit attempts with daily and non‐daily NRT use. In the sensitivity analysis that collapsed daily and non‐daily use, e‐cigarette use remained associated with quit attempts (OR = 1.35, 95% CI = 1.03–1.77, *P* = 0.03).

### Smoking cessation

Among smokers not using e‐cigarettes at baseline, 168 (12.9%) quit smoking, compared with 25 non‐daily users (9.5%) and seven daily users (8.1%). Sample characteristics are presented in Table 1. Unadjusted results showed no significant association with cessation for daily (OR = 0.60, 95% CI = 0.27–1.32, *P* = 0.21) or non‐daily e‐cigarette use (OR = 0.71, 95% CI = 0.46–1.11, *P* = 0.13) compared with non‐use.

While adjusting for baseline characteristics, neither daily nor non‐daily use of e‐cigarette at baseline was associated with cessation at follow‐up and nor was NRT use (Table 1). Considering any e‐cigarette use (daily and non‐daily), we found non‐significantly reduced cessation (adjusted OR = 0.73, 95% CI = 0.48–1.09, *P* = 0.13).

### Reduction in tobacco cigarette consumption

Overall, 6.2% (*n* = 65) of respondents reduced their consumption substantially. Forty‐four (5.7%) smokers not using e‐cigarettes at follow‐up, 11 (5.5%) non‐daily e‐cigarette users and 10 (13.9%) daily users reduced substantially. Sample characteristics are included in Table 2. In unadjusted analysis of substantial reduction, daily use of e‐cigarettes at follow‐up compared with non‐use was associated with increased likelihood of reduction (OR = 2.66, 95% CI = 1.28–5.54, *P* = 0.009); non‐daily use was not associated with substantial reduction (OR = 0.96, 95% CI = 0.48–1.89, *P* = 0.90).

**Table 2:** Logistic regression analyses of association of socio‐demographics, dependence (SUTS) and non‐cigarette nicotine intake at follow‐up with substantial reduction in cigarettes per day (CPD).

		*Reduction (n = 1042, of whom n = 65 reduced CPD by ≥50% of baseline)*			
		*n(%) /mean (SD)*	*OR*	*95% CI*	*P*
Baseline age^a^		46.7 (15.3)	0.99	0.78 to 1.08	0.30
Gender	Female	455 (43.7)	1		
	Male	587 (56.3)	0.51	0.30 to 0.86	0.012
Baseline level of education	No HE	706 (67.8)	1		
	Some HE	336 (32.3)	0.90	0.52 to 1.57	0.71
Baseline SUTS^b^		2.1 (1.1)	0.76	0.59 to 0.98	0.031
Follow‐up NRT use	None	909 (87.2)	1		
	Non‐daily	83 (8.0)	1.50	0.61 to 3.70	0.38
	Daily	50 (4.8)	1.66	0.58 to 4.70	0.34
Follow‐up e‐cig use	None	769 (73.8)	1		
	Non‐daily	201 (19.3)	0.85	0.43 to 1.71	0.66
	Daily	72 (6.9)	2.49	1.14 to 5.45	0.022

a Mean and standard deviation (SD) presented, odds ratio (OR) for single year raised to the power of 10 to present per 10‐year increase.

b Strengths of urges to smoke, possible range 0 ‘no urges’ to 5 ‘extremely strong urges’, mean and SD presented, OR per unit increase. HE = higher education NRT = nicotine replacement therapy.

In the primary reduction analysis and while adjusting for other relevant characteristics, daily use of e‐cigarettes remained associated with increased likelihood of reduction while non‐daily use was not associated significantly with substantial reduction (Table 2). Neither daily nor non‐daily NRT use was associated with substantial reduction (Table 2).

When daily and non‐daily e‐cigarette use were collapsed, this was not significantly different from non‐use (OR = 1.23, 95% CI = 0.70–2.15, *P* = 0.48). Secondary analysis in those using e‐cigarettes at both time‐points, adjusted for the same variables as the primary analysis, showed that compared with non‐use at follow‐up (*n* = 769), daily e‐cigarette use (*n* = 79) was again associated with substantial reduction (OR = 4.19, 95% CI = 2.13–8.24, *P* < 0.001), while non‐daily use (*n* = 157) was not (OR = 1.02, 95% CI = 0.48–2.19, *P* = 0.96).

Linear regression on quantitative change in CPD indicated that the difference in change between those using e‐cigarettes daily and those not using them at follow‐up (Table 3) was significant while adjusting for baseline age, gender, education, dependence and follow‐up NRT use {[B [standard error (SE)] = –1.55 (0.65), β = –0.08, *P* = 0.02}. The difference in change between non‐daily users and non‐users was not significant [B (SE) = 0.28 (0.41), β = 0.02, *P* = 0.50] Secondary analysis in those using e‐cigarettes at both time‐points suggested a larger difference between changes for daily users and non‐users [Table 3, B (SE) = –2.58 (0.61), β = –0.14, *P* < 0.001, adjusted as before], whereas the difference in change between non‐daily users and non‐users remained small [B (SE) = –0.08 (0.44), β = –0.01, *P* = 0.85].

**Table 3:** Cigarettes per day by frequency of e‐cigarette use.

	*Mean (SD) cigarettes per day*		
*Follow‐up e‐cigarette use*	*Baseline*	*Follow‐up*	*Change*
None	13.3 (8.9)	13.5 (8.9)	0.2 (4.7)
Primary analysis, use initiated after baseline			
Non‐daily	13.5 (7.9)	13.9 (8.9)	0.4 (5.9)
Daily	14.3 (9.8)	13.0 (9.4)	–1.4 (6.8)
Secondary analysis, some use at baseline			
Non‐daily	14.9 (8.9)	15.0 (8.0)	0.09 (5.4)
Daily	14.1 (7.9)	11.5 (7.2)	–2.5 (6.1)

SD = standard deviation.

## Discussion

In a web‐based national sample of smokers from the general population, those using e‐cigarettes daily at baseline were more likely to have attempted to stop smoking when followed‐up a year later than smokers not using e‐cigarettes, but neither non‐daily nor daily e‐cigarette use was associated with smoking cessation during follow‐up. Smokers using e‐cigarettes daily when followed‐up were more likely to have achieved at least 50% reduction in tobacco cigarette consumption from baseline. Less frequent e‐cigarette use did not have a significant effect on consumption. Using e‐cigarettes every day while smoking increased the prevalence of substantial reduction in tobacco consumption, and this was not restricted to smokers who had recently taken up e‐cigarettes, suggesting that persistent users continue to reduce consumption over time. Reduction in consumption has been reported previously 14. This increase in substantial reduction was reflected in a small overall reduction in the number of cigarettes smoked in daily e‐cigarette users. The size of the reduction was similar to that seen in smokers using NRT 12. NRT itself showed a similar size of positive association with subsequent cessation to that found in previous studies 12, but in this case it was not statistically significant using a conventional alpha (*P* = 0.067 two‐tailed).

The use of NRT while smoking is supported as a harm reduction approach by national guidance in the United Kingdom 24. It reduces tobacco harm not only by increasing cessation and reducing consumption but also by reducing the amount of nicotine taken in from each cigarette 25, which is likely to be accompanied by a reduction in intake of toxins 26, 27. Although it remains to be tested, it appears possible that the use of e‐cigarettes while smoking similarly reduces intake from each cigarette, thus supporting tobacco harm reduction. Although long‐term data on safety of e‐cigarettes are not yet available, toxicology testing suggests that they will be considerably safer than tobacco cigarettes 28, although they may be less safe than NRT, which is licensed as medicine.

Smoking cessation rates in England were higher in 2014 than in previous years. Generally, cessation rates in a population can be increased by encouraging as many smokers as possible to make quit attempts and to use the most effective support in each of these attempts. The current data indicate that e‐cigarettes were associated with more smokers attempting to stop smoking. We found no evidence that e‐cigarette use while smoking increased subsequent smoking cessation. This is in line with previous findings 9, 15, 16, although in one recent study intense long‐term use was associated with increased cessation 17. The present analyses extend the evidence by assessing use prospectively, thus avoiding confounding with quit attempts (otherwise e‐cigarette use may be mainly a marker of having made a quit attempt) and by assessing quit attempts, cessation and reduction separately. Further research on the link between smoking cessation rates and e‐cigarette use is warranted.

Importantly, the current sample used e‐cigarettes for any reason, not necessarily to stop smoking, so the results cannot be used to derive statements on their effectiveness as cessation aids. Few studies have looked at e‐cigarettes as cessation aids. One randomized controlled trial indicated that the particular e‐cigarette used in the trial was of similar effectiveness as nicotine patches in supporting abstinence 29. Use and effects of different devices in the general population are likely to differ from those in controlled trials and samples of dedicated e‐cigarette users may differ from other users in the general population. A recent study using a representative population sample found that smokers who used e‐cigarettes in an attempt to stop smoking were more likely to report continued abstinence than those using NRT without prescription or no aids 30. Further high‐quality longitudinal studies are needed on e‐cigarettes as cessation aids. Future research should also evaluate the impact of continued use of e‐cigarettes on smoking behaviour, as we were only able to provide snapshots of use at two time‐points.

Further evidence is needed on differences between the numerous types of e‐cigarettes, as products vary widely in their appearance, function, content, marketing and nicotine delivery 31, 32, 33, 34, and use and effects on smoking will vary considerably across different types. In this sample, the majority were using cigarette‐like products. These have been found to deliver less nicotine than more recently developed products 22, 32, 35, and in a sample of ex‐smokers who had quit using e‐cigarettes all had used more recently developed products 36, indicating that cigarette‐like e‐cigarettes may be less helpful.

Several limitations of the study should be noted. Follow‐up rate was 43%, resulting in small sample sizes for some analysis. Respondents who were followed‐up differed from those not followed‐up on some demographic variables, specifically age and gender, potentially reducing the generalizability to younger and female smokers. However, key smoking characteristics and e‐cigarette use were not associated with follow‐up. The survey did not include questions on the duration of use, so non‐daily e‐cigarette users will have included people who had just tried e‐cigarettes once or twice, as well as occasional users. This also means that we did not assess if respondents continued to use e‐cigarettes throughout the follow‐up period and not all baseline users may have continued to use them. Also, those initiating e‐cigarette use during the follow‐up period were included with baseline non‐users. Any short‐term use of e‐cigarettes around baseline and uptake during follow‐up will therefore have led to an underestimation of their effects on quit attempts and cessation. Additionally, the baseline sample including only smokers would have excluded any ex‐smokers who had used e‐cigarettes and successfully quit, thus potentially biasing the sample in favour of ‘treatment failures’. The definition of cessation did not include a minimum time of abstinence, but relied upon respondents’ self‐report. However, this method avoids recall bias, and in population surveys the risk of misreporting is reduced, as there is no expectation to report cessation 37. The online recruitment method is likely to have led to some selection bias, as internet use is linked to socio‐economic status and age; however, the socio‐economic divide has narrowed considerably between 2011 and 2013 38. The sample was self‐selected in so far as participants had volunteered for a market research company web panel; nevertheless, the overall sample characteristics were broadly similar to those of representative samples from a national household survey 22, 39.

The recruitment method also represents a strength, as in contrast to many early studies of e‐cigarettes that recruited from e‐cigarette interest groups (e.g. 33, 40), recruitment was not from self‐selected populations with decidedly positive attitudes towards the devices. Thus, the association between their use and changes in smoking behaviour found in this study is expected to be more widely generalizable. The present survey has overcome another limitation of the very small number of previous longitudinal studies by separating regular and occasional use. More frequent use showed an effect on smoking behaviour where occasional use did not, and the effect of e‐cigarettes on reduction in tobacco consumption disappeared when not differentiating frequency of use, suggesting that previous analyses may have overlooked effects. The current study may have missed important factors associated with quit attempts, cessation or reduction; for example, the use of other aids to stop smoking or mental health status of respondents. However, by adjusting for a range of important characteristics such as age, gender and dependence, it takes into account more potential confounders than previous longitudinal studies 15, 40.

## Conclusions

Daily use of e‐cigarette use while smoking at one time‐point is associated with subsequent increases in rates of attempting to quit smoking and reducing smoking, but not with increased smoking cessation. These effects persisted after adjusting for a range of socio‐demographic characteristics, dependence and other nicotine use. Non‐daily use of e‐cigarettes while smoking is not associated with quit attempts, cessation or reduced smoking. These findings illustrate the importance of differentiating ever use or very occasional use from regular use in assessing the effects of e‐cigarette use on smoking behaviour, a differentiation that has frequently been overlooked in previous research.

## Declaration of interests

J.B. has received an unrestricted grant from Pfizer, R.W. undertakes research and consultancy and receives fees for speaking from companies that develop and manufacture smoking cessation aids (Pfizer, J&J, McNeil, GSK, Nabi, Novartis and Sanofi‐Aventis). L.B., S.H. and A.M. have no relationships with companies that might have an interest in the submitted work. There are no other financial relationships with any organizations that might have an interest in the submitted work, particularly electronic cigarette companies, and there are no other relationships or activities that could appear to have influenced the submitted work.

## Supporting information




**Appendix S1** Measures
**Table A1** Comparison of respondents followed up and lost to follow‐up

Supporting info item
