# Supplementary material for: Is the use of electronic cigarettes while smoking associated with smoking cessation attempts, cessation and reduced cigarette consumption? A survey with a 1‐year follow‐up
Source: Addiction. 2015 Apr 23;110(7):1160–8. doi: 10.1111/add.12917 (PMC4862028; doi:10.1111/add.12917)
Supplement: Supplementary file 1 — Supporting info item [file ADD-110-1160-s001.docx]

**Appendix. Measures**

**Highest level of education**

What is the highest level of formal education that you have completed?

1. Primary or secondary school/vocational level 1 & 2/ trade apprenticeship
2. Secondary school advanced/vocational level 3
3. Further education/training college below degree level
4. Some university
5. Completed university degree
6. Post-graduate degree
7. Don’t know
8. Prefer not to say

**Smoking status**

Which of the following best applies to you?

a) I smoke cigarettes (including hand-rolled) everyday

b) I smoke cigarettes (including hand-rolled) but not every day

c) I do not smoke cigarettes at all but I do smoke tobacco of some kind (e.g. pipe or cigar)

d) I have stopped smoking completely in the last year

e) I stopped smoking more than a year ago

f) Don’t know/ couldn’t say .

[Those responding ‘Yes’ to either (a) or (b) were classified as current (daily and non-daily) smokers and those responding ‘Yes’ to (d) or (e) were classified as ex-smokers. Those responding (c) or (f) at either wave were not included in these analyses. ]

**Quit attempts**

How many serious attempts to stop smoking have you made in the last 12 months? By serious attempt we mean you decided that you would try to make sure you never smoked again. Please include any attempt that you are currently making and please include any successful or unsuccessful attempt made within the last year.

- ….times
- Don’t know/couldn’t say

**Tobacco cigarette consumption**

How many cigarettes do/did you usually smoke? We are just looking for your best estimate.

- Please enter number per day if usually smoke/d every day
- Please enter number per week if you do/did not usually smoke every day
- Don’t know / couldn’t say

**Strengths of urges to smoke**

1) How much of the time have you felt the urge to smoke in the past 24 hours?

1. Not at all
2. A little of the time
3. Some of the time
4. A lot of the time
5. Almost all of the time
6. All of the time

2) In general, how strong have the urges to smoke been?

1. Slight
2. Moderate
3. Strong
4. Very strong
5. Extremely strong

[Question 2 is asked of those choosing a response other than a) for question 1. Those who chose response a for question 1 were coded as 0 to create a final strength of urges scale ranging from 0 (no urges) to 5 (extremely strong urges).]

**Nicotine replacement therapy use**

Can I check, are you using any of the following either to help you stop smoking, to help you cut down or for any other reason at all? [Positive response to a, b, c , d, e or g coded as using NRT.]

1. Nicotine gum
2. Nicotine replacement lozenges\tablets
3. Nicotine replacement inhaler\inhalator
4. Nicotine replacement nasal spray
5. Nicotine patch
6. Electronic cigarette
7. Nicotine mouthspray
8. Another nicotine product
9. Other (please write in)
10. None of these / not using anything
11. Don’t know

How often on average do you use your nicotine replacement product or products? [Asked if any used]

1. Every day
2. Not every day, but at least once a week
3. Less often than once a week
4. Don’t know / couldn’t say

**E-cigarette use**

How often, if at all, do you currently use an electronic cigarette? [Asked of respondents who had ever heard of e-cigarettes and had ever tried one.]

1. Daily
2. Less than daily, but at least once a week
3. Less than weekly, but at least once a month
4. Less than monthly
5. Not at all
6. Don't know

**Type of e-cigarette used**

Three questions were used to determine type, responses to questions 2 and 3 were checked against information from producers and retailers of e-cigarettes to determine whether respondents were using cigalikes or other types ([23](#_ENREF_23)).

1) What electronic cigarette equipment do you currently use the most?

1. A disposable electronic cigarette (non-rechargeable)
2. A commercial electronic cigarette kit which is refillable with pre-filled cartridges
3. A commercial electronic cigarette kit which is refillable with liquids
4. A modular system (I use my own combination of separate devices: batteries, atomizers, etc.)
5. Don’t know

2) What is the name of the brand of e-cigarettes that you currently use the most?

Comprehensive list of brands and choice of ‘other brand (please write in)’.

3) What is the name or type of e-cigarette product that you use in their range? Please try and be as specific as possible. For example, someone using E-Lites may indicate that they are using the ‘E40’ model in that range, with a ‘Regular/Red’ nicotine strength. This is the level of detail that we are ideally looking for.

Free-text responses

**Table A1. Comparison of respondents followed up and lost to follow-up**

| **Baseline characteristics** | | **Followed up, n=2182** | **Lost to follow-up, n=2818** | **Comparison statistic** |
| --- | --- | --- | --- | --- |
| **Age, M (SD)** |  | 46.4 (15.5) | 40.8 (14.4) | t=13.3, p<0.001 |
| **Gender, % female** |  | 41.8 | 51.7 | χ^2^=48.8, p<0.001 |
| **Education, % no higher education** |  | 63.7 | 61.6 | χ^2^=2.3 , p=0.13 |
| **SUTS, M (SD)*** |  | 1.9 (1.2) | 2.0 (1.2) | t=1.73, p=0.08 |
| **NRT use, %** | None | 82.0 | 77.6 | χ^2^=13.8 , p=0.001 |
|  | Non-daily | 10.6 | 13.7 |  |
|  | Daily | 7.4 | 8.3 |  |
|  | Missing (don’t know) | 0.2 | 0.4 |  |
| **E-cigarette use, %** | None | 80.8 | 78.1 | χ^2^=5.4 , p=0.07 |
|  | Non-daily | 14.0 | 15.9 |  |
|  | Daily | 5.1 | 5.9 |  |
|  | Missing (don’t know) | 0.1 | 0.1 |  |

* N=81 reported don’t know in response to either of the questions from which the SUTS is derived. Of those, 51 (63%) were lost to follow-up
